# The causes and consequences of trained immunity in myeloid cells

**DOI:** 10.3389/fimmu.2024.1365127

**Published:** 2024-04-11

**Authors:** Gunapati Bhargavi, Selvakumar Subbian

**Affiliations:** Public Health Research Institute, New Jersey Medical School, Rutgers University, Newark, NJ, United States

**Keywords:** inflammation, macrophage, neutrophil, epigenetics, metabolism, cell signaling, innate immunity, animal models

## Abstract

Conventionally, immunity in humans has been classified as innate and adaptive, with the concept that only the latter type has an immunological memory/recall response against specific antigens or pathogens. Recently, a new concept of trained immunity (a.k.a. innate memory response) has emerged. According to this concept, innate immune cells can exhibit enhanced responsiveness to subsequent challenges, after initial stimulation with antigen/pathogen. Thus, trained immunity enables the innate immune cells to respond robustly and non-specifically through exposure or re-exposure to antigens/infections or vaccines, providing enhanced resistance to unrelated pathogens or reduced infection severity. For example, individuals vaccinated with BCG to protect against tuberculosis were also protected from malaria and SARS-CoV-2 infections. Epigenetic modifications such as histone acetylation and metabolic reprogramming (e.g. shift towards glycolysis) and their inter-linked regulations are the key factors underpinning the immune activation of trained cells. The integrated metabolic and epigenetic rewiring generates sufficient metabolic intermediates, which is crucial to meet the energy demand required to produce proinflammatory and antimicrobial responses by the trained cells. These factors also determine the efficacy and durability of trained immunity. Importantly, the signaling pathways and regulatory molecules of trained immunity can be harnessed as potential targets for developing novel intervention strategies, such as better vaccines and immunotherapies against infectious (e.g., sepsis) and non-infectious (e.g., cancer) diseases. However, aberrant inflammation caused by inappropriate onset of trained immunity can lead to severe autoimmune pathological consequences, (e.g., systemic sclerosis and granulomatosis). In this review, we provide an overview of conventional innate and adaptive immunity and summarize various mechanistic factors associated with the onset and regulation of trained immunity, focusing on immunologic, metabolic, and epigenetic changes in myeloid cells. This review underscores the transformative potential of trained immunity in immunology, paving the way for developing novel therapeutic strategies for various infectious and non-infectious diseases that leverage innate immune memory.

## 1 Background

Immunity conferred by the cells of the host immune system represents the main line of defense that recognizes, responds, and protects against invading pathogens such as bacteria, viruses, fungi, and other foreign particles (1). Conventionally, immunity in humans is classified as innate and adaptive responses. The innate immunity prevails from the neonatal stage, which protects the host non-specifically against a wide range of pathogens. Components of the innate immune response include natural barriers of the body, such as skin, and mucous as well as cellular chemical barriers, such as enzymes and antimicrobial molecules. Innate immune cells include phagocytes, such as macrophages, dendritic cells (DC), neutrophils, and non-phagocytic cells, including natural killer (NK) cells and gamma-delta T-lymphocytes (2). In contrast to innate immunity, adaptive immunity develops over time and is specific to pathogens and/or their components, with additional active and passive immunity features (3). The primary cells of adaptive immunity are the T and B lymphocytes, with several subtypes within these two classes of cells. While active immunity develops after exposure to a pathogen or vaccination, allowing the body to produce its own antibodies and memory cells for long-term protection, passive immunity is mediated by existing antibodies providing temporary protection against infection (4, 5).

**Figure 1:**
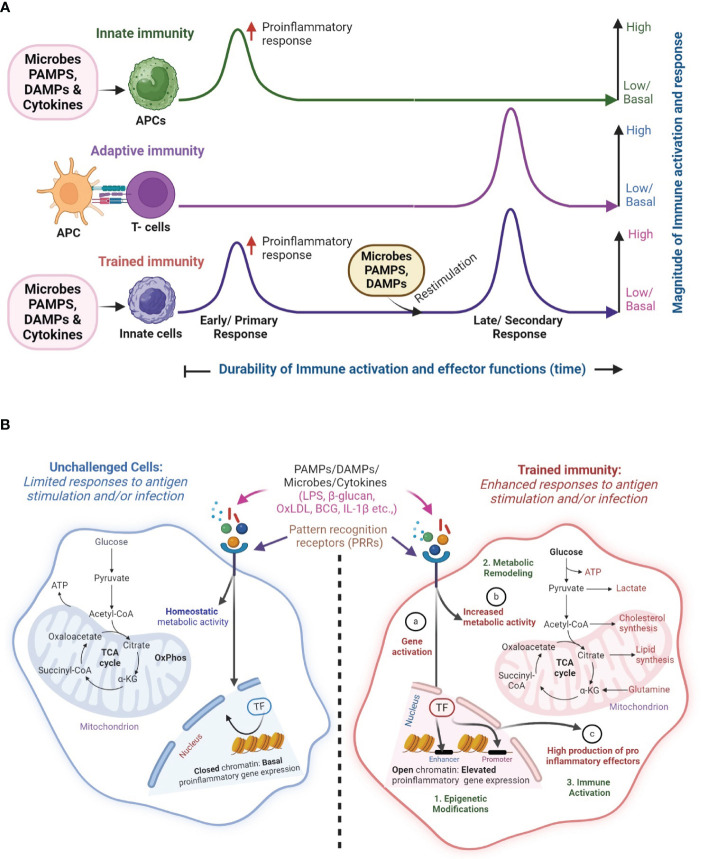
Overview of classical innate, adaptive and trained immunity. **(A)** In classical innate immunity, innate immune cells such as macrophages recognize microbes, pathogen-associated molecular patterns (PAMPs), host-derived danger-associated molecular patterns (DAMP) or cytokines through their cell surface pattern recognition receptors (PRRs) and elicit a primary response, which mostly results in a non-specific early proinflammatory response. In contrast, during adaptive immunity, T cells are activated by specific signaling from antigen-presenting cells (APCs). Upon restimulation with PAMPs and DAMPs, the memory T cells exert a robust immune response in an antigen-specific manner. This memory (recall) response is the classical hallmark of adaptive immune response. However, the memory-like response of innate immune cells, secondary to the innate immunity, upon restimulation of PAMPs and DAMPs is the hallmark of trained immunity, which includes the characteristics of both classical innate and adaptive immunities. **(B)** The concept of trained immunity. In unchallenged/non-trained innate immune cells (left; blue color), engagement of PRRs by PAMPs (e.g. LPS, β-glucan), DAMPs (e.g. OxLDL) or cytokines such as IL-1β, results in a limited immune response with homeostatic levels of metabolic, proinflammatory responses and effector gene expression, which is due to closed chromatin confirmation. However, upon restimulation, these innate immune cells elicit a robust proinflammatory response upon engagement of their PPRs by microbes, PAMPs, and DAMPs, in an antigen-independent manner. Activation of trained immunity manifests in gene activation changes through epigenetic modifications **(a)** increased metabolic activity **(b)** and elevated production of proinflammatory effector molecules **(c)** in the innate immune cells. These interrelated processes result in **(1)** epigenetic modifications, and **(2)** metabolic remodeling that culminates in **(3)** immune activation of trained cells. The image was created in BioRender.

An important feature that distinguishes adaptive immunity from innate immunity is that the former type of immunity has a memory or re-call response against specific antigens or pathogens, while the latter immunity does not have a memory response. However, a new concept in host immune response has recently been proposed, namely the trained immunity (a.k.a. innate memory response) (6). The main tenant of the trained immunity concept is that innate immune cells such as macrophages as well as non-immune cells, including epithelial and endothelial cells can develop a memory response upon stimulation with antigen/pathogen (**Figure 1A**). Thus, trained immunity enables the innate immune cells to remember and respond robustly to previous pathogens encounters (7, 8). Trained immunity is enhanced through exposure or re-exposure to infections, vaccines, or other immune-stimulating agents (9). However, these memory-like responses associated with innate immune cells are not as specific or durable as the conventional immune memory established in the cells of adaptive immunity (10).

Prior to the proposal of the trained immunity concept, few studies have reported the non-specific protective efficacy of some of the bacteria against secondary infection by a different pathogen. For example, in 1968 Mackaness et al. reported that mice inoculated with Bacillus Calmette-Guerin (BCG), a live attenuated vaccine strain of *Mycobacterium bovis*, protected the animals against a secondary infection by a pathogenic strain of *Mycobacterium tuberculosis* (Mtb) (11). In another study, the same group reported that BCG vaccination protected mice against infection by several unrelated pathogens, including *Salmonella typhimurium* and *Listeria monocytogenes* (12). Furthermore, in the early 1900s, BCG vaccination was demonstrated to reduce the general morbidity in children due to infectious diseases (13). Based on these observational studies, scientists such as Calmette and Naslund suggested that BCG vaccination can confer non-specific protection against infectious diseases other than tuberculosis (TB), which is the intended target disease of the BCG vaccine (14, 15). Built upon these observations, the concept of trained immunity was proposed by Mihai Netea in 2011 (16). Subsequently, many researchers have provided experimental evidence to explain the molecular and cellular mechanisms underpinning trained immunity, including the epigenetic and metabolic modifications in the trained innate immune cells (17, 18). In this review, we provide an overview of conventional innate and adaptive immunity and summarize various mechanistic factors associated with the onset and regulation of trained immunity, focusing on immunologic, metabolic, and epigenetic changes in innate immune cells. We also discuss the potential applications and limitations in translating trained immunity for clinical applications.

## 2 Main text

### 2.1 The innate and adaptive immune system in higher vertebrates

In response to the entry of any microbes, the innate and adaptive immune systems of the body recognize, destroy, and eliminate the invading organism through phagocytosis, without significant injury to the host (19). In general, innate immunity is the first line of defense that recognizes the pathogen through phagocytic pathogen recognition receptors (PRRs) on its surface, which bind with the surface molecules of pathogens, namely the pathogen-associated molecular patterns (PAMPs), or the host-derived danger-associated molecular patterns (DAMPs). The innate immune cells include monocytes, neutrophils, DCs and macrophages, mast cells, basophils, eosinophils, and NK cells. Among these cells, phagocytes, such as neutrophils and macrophages are mainly seen in infected tissues and are involved in engulfing/phagocytosing, degrading, and clearing the microbial- and host-cell-derived debris (20). Innate immune cells, such as macrophages express PRRs, such as Toll-like receptors (TLRs) C-type lectin receptors (CLRs), and NOD-like receptors (NLRs), which recognize PAMPs derived from pathogens, including, bacterial lipopolysaccharides (LPS), viral nucleic acids, and fungal cell wall components as well as DAMPs, which are endogenous molecules released from damaged or stressed host cells. Although PRRs exhibit broad specificity and recognize a range of both PAMPs and DAMPs, the response of phagocytes to various PAMPs and DAMPs is context-dependent (20–23). For example, TLRs, located on cell surfaces can recognize various PAMPs and initiate immune responses, while NLRs, found in the cytoplasm, form inflammasomes upon detecting PAMPs or DAMPs, leading to cytokine production (21–23). Furthermore, while PAMPs are mostly invariant antigens within a class of microbial agents, they are distinguishable from DAMPs, which are “danger” signaling molecules produced by the host because of inflammation, infection, and/or cell/tissue damage (21). Importantly, the interaction of PRRs with PAMP or DAMP activates phagocytosis and antigen processing within the phagocytes. Activated phagocytes can also migrate to regional draining lymph nodes to “present” the antigen to T cells of the adaptive immune response, through cognitive major histocompatibility complex (MHC) molecules (21). Hence, phagocytes are also termed as antigen-presenting cells (APCs). Upon activation, APCs produce pro-inflammatory cytokines and chemokines, leading to the recruitment and activation of other immune cells, including T and B lymphocytes, which results in the production of antibodies and cytotoxic T-cell activation (21–23). Thus, PRRs serve as a vital signaling link between the innate and adaptive immune cells.

Innate immunity also encompasses a complement system as an efficient defense mechanism against pathogens, contributing to pathogen clearance, inflammation, and modulation of adaptive immune responses through a complex network of soluble proteins like C3b. These proteins aid in the opsonization of microbes by binding to their surface and enhancing the phagocytosis leading to the secretion of inflammatory mediators, formation of the membrane attack complex for pathogen lysis as well as direct microbial killing, and clearing the apoptotic cells and immune complexes, thus regulating the host immune responses (24). Additionally, complement activation enhances adaptive immunity by promoting antigen presentation and antibody production. Overall, the complement system provides a rapid and effective defense mechanism against invading pathogens while regulating immune responses to maintain tissue homeostasis.

In addition to phagocytes, the innate immune system also contains non-phagocytic cells, such as mast cells, basophils, eosinophils, and NK cells. Among these, mast cells, basophils, and eosinophils are granular immune cells, that generate inflammatory responses via histamine release. It should be noted that these responses are part of the body’s defense mechanism that can sometimes contribute to disease pathology, such as in allergic reactions (25). The NK cells are a subset of cytotoxic cells of the innate immune system that mainly recognize infected cells via specific receptors, lysing and enabling them to be eliminated effectively, as recently shown for lethal CMV viremia or transcriptional control of HIV-1 (26–28). Thus, innate immunity has a significant role in protecting the host system from external agents in a non-specific manner; however, innate immune cells, in general, do not effectively function as classical memory cells to actively mount an immune response upon re-encountering the same PAMPs. While innate immunity lacks conventional cellular memory, phenomena such as trained immunity suggest a form of non-specific memory response in innate immune cells.

The adaptive immune response is also effective in eliminating pathogens, similar to innate immunity but in a different perspective (19, 29). Activation of adaptive immunity eliminates pathogens along with their toxic molecules, mostly by generating a memory response to specific PAMPs of the pathogens (30). Adaptive immunity is further categorized into humoral, and cell-mediated immunity. Humoral immunity is mediated by B lymphocytes (B cells), with a unique antigen receptor on its surface, known as the B cell receptor (BCR). The BCR is a membrane-bound immunoglobulin molecule, that recognizes specific epitopes or antigens. Affinity maturation is a process that refines the specificity and affinity of antibodies resulting in the production of antibodies that neutralize the pathogen and its secreted toxins leading to their elimination (31). The antigen-specific response is the hallmark of adaptive immunity, which refers to the ability of lymphocytes to recognize and respond to specific antigens, ensuring targeted immune responses against pathogens. On the other hand, affinity maturation, primarily occurring in B cell responses, involves the refinement of antibody binding affinity to antigens through somatic hypermutation. This process leads to the generation of antibodies with progressively higher affinity, enhancing the effectiveness of the immune response over time. Together, antigen specificity and affinity maturation are fundamental aspects of the adaptive immune system’s ability to mount precise and potent responses tailored to encountered antigens.

In addition to mounting an immediate, antigen-specific effector function, the adaptive immune system develops long-term memory responses through the formation of memory T and B cells during the primary immune response. These memory cells remain quiescent but quickly respond upon re-exposure to the same pathogen, leading to a faster and more potent secondary immune response. This memory provides long-lasting immunity against previously encountered pathogens (31). Thus, adaptive immunity advances to target pathogens more effectively through processes such as antigen presentation, clonal selection, affinity maturation, and memory cell formation. While APCs initiate adaptive immune responses by presenting antigens to T and B cells, which then undergo clonal selection, leading to the expansion of cells targeting the pathogen, the affinity maturation fine-tunes those responses by generating antibodies with higher affinity for the antigen (32). Further differentiation of adaptive immune cells into effector cells aids in coordinating and executing immune responses, and finally, the memory cell formation ensures rapid and potent responses upon re-exposure to the same pathogen. Thus, adaptive immunity continuously evolves to mount faster, more specific, and more effective responses against pathogens.

### 2.2 Trained immunity

Trained immunity (a.k.a. Innate memory response) is an emerging branch of host immune response that defines the ability of innate immune cells to generate non-specific immunological memory responses, which can confer long-term protection against infections (**Figure 1A**). Trained immunity and innate immunity provide broad, non-specific protection against a range of pathogens. Innate immunity encompasses the broader array of non-specific defense mechanisms present from birth, whereas trained immunity specifically refers to the enhanced responsiveness of innate immune cells following exposure to certain stimuli. Similarly, while adaptive immunity is marked by a precise, antigen-specific recall response, the hallmark of trained immunity is its non-specific and antigen-independent immune response. Trained immunity is induced by innate immune cells, such as macrophages, monocytes, and NK cells, or in case of reentry of pathogen or vaccination. The development of trained immunity occurs at a preliminary level (central training) with the involvement of the hematopoietic stem (HSCs) and progenitor cells (HSPCs) (33). These cells, residing in the bone marrow have a longer life span and respond rapidly to protect the host against chronic infections (34, 35). In addition to the bone marrow-derived HSCs and HSPCs, monocytes and granulocytes with respectively, Ly6C^+^ and Ly6G^+^/Gr-1^+^ phenotypes, are associated with the trained immunity-mediated effector functions, including degranulation and release of proinflammatory molecules upon infection by various pathogens (36–38). These immune responses can retain immunological memory between the self and non-self which leads to the establishment of a long-term trained immunity (7, 39).

Epidemiological observation studies in humans vaccinated with BCG, intended to protect against TB, indicated a non-specific cross-protection against sepsis and respiratory tract infections in vaccinated individuals (40). Similar observations of cross-protection were noted for the measles vaccine and smallpox vaccine against general mortality in children and leprosy or whooping cough, respectively (41, 42). Further, the existence of trained immunity has been reported in various *in vivo* studies, including treating mice with different antigens and stimulants, which would protect against infection. For instance, administration of β-glucan, a fungal cell-surface ligand protected normal and leukemic mice against systemic sepsis caused by *Staphylococcus aureus* infection (43, 44). Similarly, intraperitoneal administration of CpG, an oligo deoxy nucleotide, protects against meningitis caused by *E. coli* infection in neutropenic mice models (45). A study on SCID mice that lack functional T cells and B cells showed that BCG vaccination protected the mice against *Candidiasis* (46, 47). Further, BCG vaccination in Rag^-/-^ knockout and athymic mice lacking T cells were protected against re-infection with *C. albicans* (48, 49). Similarly, β-glucan induces trained immunity in splenectomized mice, and removal of the spleen did not modulate the expression of pro-inflammatory cytokines or circulating monocytes or NK cells (50). In another study, activation of trained immunity through intraperitoneal injection of LPS and BCG was shown to be associated with the induction of inflammation and fibrosis in Balb/c mice, marked by increased expression of cytokines (IL-6, IL-1β, IL-6 and IL-10), chemokines (CCR2, CCR4, TLR-2, and TLR-4), inflammatory (Ly6C and CD43) and co-stimulatory receptors (CD80 and iCOS) by the stimulated splenocytes (51). It should be noted that in standard mice models, the contributions of innate and adaptive immunity are intertwined, making it challenging to dissect the specific roles of each component. However, the SCID and Rag-/- mice lack functional T and B lymphocytes, rendering them incapable of mounting adaptive immune responses (52–54). Therefore, SCID and Rag-/- mice allow researchers to study the role of innate immunity, including trained immunity, in host defense innate immune response, without the confounding effects of adaptive immunity. Furthermore, due to their reliance on innate immune responses, SCID and Rag-/- mice may exhibit heightened sensitivity to stimuli that induce trained immunity (52–54). This increased sensitivity can facilitate the detection of subtle changes in innate immune function and provide insights into the mechanisms underlying trained immunity. Thus, data from studies obtained in transgenic mice models that are defective in adaptive immunity, such as lack of secondary lymphoid organ (i.e. spleen) or T and B cells, highlights the functional contribution of trained immunity in mounting an effective immune response and/or protecting the infected host. However, findings from studies in SCID and Rag-/- mice should be interpreted in the context of their immunodeficient status and may not fully recapitulate immune responses in normal physiological conditions (52–54).

The immunostimulants of trained immunity, including β-glucan, oxidized low-density lipoprotein (oxLDL), and BCG induce epigenetic reprogramming through histone modifications and metabolic shifts in innate immune cells, such as macrophages. These stimuli trigger changes in proinflammatory gene expression profiles and immunometabolic pathways, enhancing immune activation and host defense mechanisms (18). For example, β-glucan activates Dectin-1 signaling, promoting glycolysis and pentose phosphate pathway while suppressing oxidative phosphorylation (OXPHOS). Similarly, oxLDL engages scavenger receptors, inducing epigenetic alterations and pro-inflammatory responses, while BCG induces epigenetic reprogramming and shifts macrophage metabolism toward glycolysis, contributing to trained immunity and improved host defense (18). An *in vitro* study on human monocytes using β-glucan, oxLDL, and BCG as stimulants, induced trained immunity with increased reactive oxygen species (ROS) production and metabolic shift towards glycolysis, which activated a proinflammatory response of these APCs (55). It should be noted that activation of trained immunity by different stimuli may lead to varied trained responses that have potential implications for disease resistance and homeostasis. For example, in patients with systemic lupus erythematosus (SLE), excessive inflammation triggered by necrotic debris of neutrophils and macrophages, including nucleic acids and proteins, impairs the innate immune functions, leading to poor phagocytosis, formation of immune activation complex (IAC) and auto-antigens (18). In addition, histone modifications were also impaired in SLE patients, which alters the epigenetic and immunometabolic reprogramming of APCs. Since these processes and pathways are directly associated with key immunological responses of trained immunity, activation of these biological functions through trained immune activation of APCs would further worsen the disease pathology, as seen in SLE patients (18). Overall, various antigenic stimuli, such as PAMPs, DAMPs, and vaccines can modulate the trained immunity of innate immune cells to different extents through the interplay between epigenetic remodeling, cellular metabolism, and immune function.

Apart from infection, physical exercise can also impact the ability of innate immune cells to develop trained immunity. Regular exercise has been shown to enhance trained immunity through various mechanisms, including improved immune cell function, anti-inflammatory effects (switching from proinflammatory M1 to anti-inflammatory M2 phenotype), metabolic adaptations (elevating mitochondrial quality and function), stress reduction, and epigenetic modifications (56, 57). These effects have significant implications for human health and disease prevention strategies, as exercise may reduce the risk of infections, autoimmune diseases, and chronic inflammatory conditions. To demonstrate the impact of trained immunity on APCs’ function in the context of exercise, C57BL/6 mice were subjected to daily exercise at 1 hour per day on a treadmill for 8 weeks, and BMDMs were isolated from these mice and stimulated with LPS to elicit trained immunity. Interestingly, BMDM from exercised mice induced a higher NF-κB activation and associated proinflammatory gene expression, compared to the control mice. This suggests that moderate exercise reprograms the metabolic pathways related to trained immunity in BMDMs (58). Thus, activation of trained immunity during regular physical activity could promote immune health and protect the host against the burden of infectious and inflammatory diseases.

Trained immunity may contribute to the pathogenesis of chronic inflammatory conditions, such as atherosclerosis, rheumatoid arthritis, and inflammatory bowel disease (7, 18). In these conditions, dysregulated trained immunity can lead to excessive inflammation and tissue damage, exacerbating disease pathology (7, 18). Conversely, modulating trained immunity through targeted interventions may offer therapeutic opportunities for managing chronic inflammation. For example, dampening trained immune responses could help mitigate inflammation in conditions like atherosclerosis, while enhancing trained immunity may promote immune surveillance and tissue repair (7, 18). In a study by Bhattarai et al, a dystrophin-deficient mice BMDM, which lacks a chemokine receptor, CCR2, was used to study the effect of trained immunity on Duchenne muscular dystrophy (DMD). In this study, stimulation of BMDMs isolated from DMD mice with β-glucan resulted in TLR-4-dependent functional and epigenetic changes, inducing a memory response with the release of pro-inflammatory markers of trained immunity (59, 60).

Similarly, trained immunity has been implicated in the pathogenesis of autoimmune diseases, in which the immune system mistakenly targets self-antigens and mounts an inflammatory response. Dysregulated trained immune responses may contribute to the breakdown of immune tolerance and the perpetuation of autoimmunity as described above for SLE (7, 18). Modulating trained immunity could be explored as a potential strategy for managing autoimmune diseases. For instance, dampening trained immune responses might help mitigate autoinflammatory processes, while enhancing regulatory mechanisms could promote immune tolerance and reduce autoimmunity (7, 18, 60).

Trained immunity also plays a critical role in host defense against microbial infections by providing a faster and more robust immune response. Vaccines, such as BCG, measles, and monkeypox can induce trained immunity and enhance protection against unrelated infections (7, 18). Leveraging trained immunity in vaccine development and immunization strategies may improve vaccine efficacy, particularly in populations with impaired adaptive immune responses, such as the elderly or immunocompromised individuals. Understanding the mechanisms underlying trained immunity in specific infectious diseases could inform the development of novel therapeutic approaches and adjuvants for enhancing immune responses and combating infections.

Trained immunity is durable and can exhibit long-lasting effects, persisting for weeks to months after the initial stimulus (7, 18, 61). This durability allows for sustained protection against infections and may contribute to vaccine efficacy. However, the duration of trained immunity responses can vary depending on factors such as the nature of the stimulus and the strength of the elicited immune response (7, 18, 62). In addition, there is considerable variability exists in the magnitude and duration of trained immunity responses among individuals. Some of the factors contributing to this variability include genetic background, age, sex, environmental exposures, and pre-existing health conditions. While trained immunity is beneficial for enhancing host defense, overactivation of innate immune responses can lead to chronic inflammation and tissue damage. Dysregulated trained immunity has been implicated in the pathogenesis of chronic inflammatory conditions, autoimmune diseases, and metabolic disorders. Therefore, understanding the factors that influence individual responses and balancing the activation of trained immunity with regulatory mechanisms is important for optimizing therapeutic interventions and vaccine strategies to prevent excessive inflammation and maintain immune homeostasis.

### 2.3 Mechanisms underlying enhanced immune responses in trained immunity

**Figure 2:**
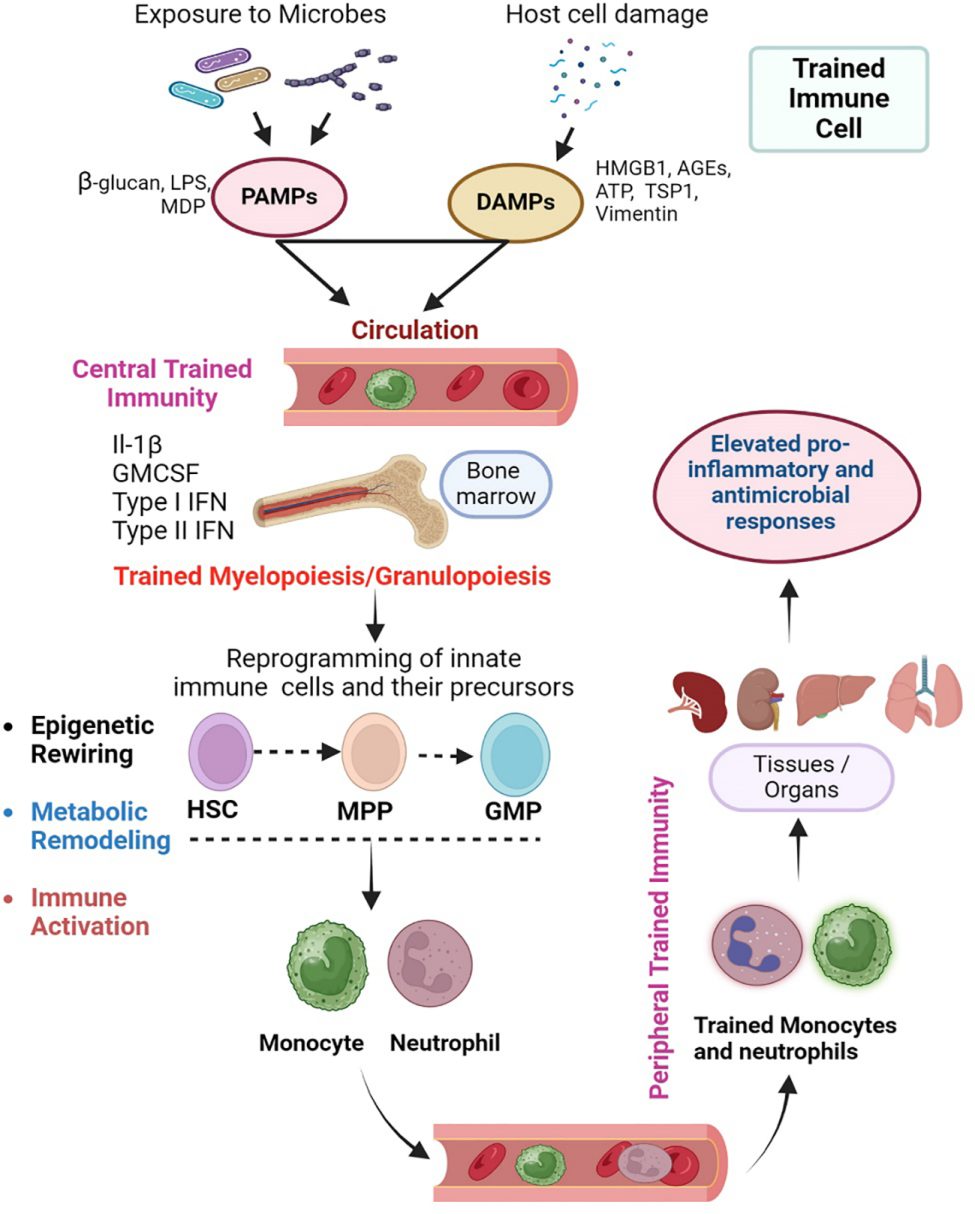
Cellular events in the orchestration of trained immunity. Trained immunity is exerted at the level of myeloid progenitor cells in the bone marrow as “central training” that further manifests as “peripheral training” in various tissues/organs. Microbes, PAMPs and DAMPs can enter the circulation and stimulate the progenitor hematopoietic stem cells (HSC) in the bone marrow. This centrally trained immunity is characterized by the differentiation of HSCs into multipotent progenitor (MPP) and granulocyte/monocyte progenitors (GMP), which ultimately produce the monocytes/macrophages and neutrophils. These centrally trained innate immune cells reach various organs through circulation, where they exert robust and rapid proinflammatory and antimicrobial responses upon stimulation of their PRRs by PAMPs or DAMP (Peripheral trained immunity). The central training of innate immune cells in the bone marrow involves an interrelated epigenetic rewiring and metabolic remodeling that ultimately leads to immune activation. The image was created in BioRender.

Since the inception of the trained immunity concept, researchers have provided experimental evidence for various mechanistic aspects of the ability of innate immune cells to develop memory responses (**Figure 1B**). Modulations in the immunological, epigenetic, and metabolic aspects of innate immune cells upon stimulation with antigens, have been implicated as the primary mechanisms in establishing trained immunity. These three mechanisms are interdependent and regulated by multiple crisscrossing cellular signaling pathways and networks that ultimately culminate in the effector response changes observed in trained innate immune cells (**Figure 2**).

#### 2.3.1 Immunological changes of trained immunity in monocytes/macrophages

Findings from *in vitro* and *in vivo* experiments suggest that trained immune cells exhibit increased phagocytosis with effective antimicrobial defense producing pro-inflammatory cytokines and chemokines that mount a rapid response to infections (63). Specifically, trained innate immune cells produce higher levels of pro-inflammatory cytokines, such as interleukin-1 β (IL-1β), interleukin-6 (IL-6), and tumor necrosis factor-alpha (TNF-α) as well as IL-15 and type I interferons (IFN), and chemokines including IL-8 and or monocyte chemoattractant protein (MCP-1) that regulates not only the migration and activation of cells at the site of infection or inflammation but can also have profound bystander effects on NK cell activation (27, 28, 64, 65). Trained phagocytes with reinforced microbicidal activity are better equipped to kill pathogens by generating ROS and reactive nitrogen species (RNS), that are toxic to bacteria, viruses, and other pathogens (66). In addition, trained APCs, such as dendritic cells can process and represent antigens quickly and efficiently to T cells through the MHC class 1 and 2 pathways. This leads to the activation of adaptative immune responses, which generate the antigen-specific memory T cells and can confer cross-protection against a wide range of infections (67). Thus, although trained immunity is primarily associated with innate immune cells, it can also influence the development of adaptive immune memory (68). Since trained immunity is non-specific, cells of the trained immunity can cross protection meaning exposure to one pathogen can enhance the immune response to unrelated pathogens. For example, the BCG vaccine, mainly used to prevent TB, can protect against other bacterial and viral infections (69). Both the microbial PAMPs, including LPS, β-glucan, muramyl dipeptide (MDP), and ligands derived from bacterial, fungal, and viral pathogens, as well as DAMPs such as lipoproteins, uric acid, and heme, can stimulate the training of innate immune cells through respective PRRs, including TLRs. NLRs and CLRs (70). PRR engagement triggers downstream signaling cascades, including activation of NF-κB, MAPK, and IRF transcription factors, which induce the expression of pro-inflammatory cytokines including TNF-α, IL-1β, and IL-6 (6–10, 18). The outcome of trained immunity following the interaction of a stimulant with innate immune cells is determined by the nature of the stimuli, the amount of the stimulus, and the duration of the interaction (71, 72). For example, at lower concentrations, stimulation of monocytes with LPS or flagellin has been shown to induce trained immunity, while at higher doses, these stimulants induce an opposite effect, namely immune tolerance (8, 71, 73, 74). Thus, different doses of the same stimulants can differentially activate the immunologic signaling pathways, leading to diverse outcomes in the trained macrophage response. A study reported that priming of PBMCs isolated from healthy donors with β- glucan elevated the production of proinflammatory cytokines, including TNF-α and IL-6 (75). However, these monocytes when pre-stimulated with LPS before β- glucan stimulation induced tolerance without induction of proinflammatory marker expression. In this study, the monocyte training was associated with the P38 and JNK-mediated MAPK signaling pathway, which directed the differential functional fate of the trained monocytes. Interestingly these monocyte’s long-term effector functions were associated with epigenetic modifications, such as histone methylation and acetylation (76, 77).

Apart from the dose of the stimulant, the biological sex also appears to differentially impact the onset of trained immunity in macrophages. In a study by Sun et al, treatment with 17β estradiol has been shown to promote trained immunity mainly in female mice against sepsis, and the mechanism underpinning this trained immunity has been postulated to be the macrophage polarization through nucleus translocation of RelB, a transcriptional regulator and member of the NFκB signaling pathway (78). Therefore, the nature of the trained immune response differs depending on the stimulants, and trained immunity regulates either tolerance or immune effector functions (8, 78–80).

#### 2.3.2 Epigenetic changes in trained monocytes and macrophages

One of the crucial processes that reprogram innate immune cells such as macrophages and monocytes during trained immunity is epigenetic remodeling, which includes histone modifications by histone acetyltransferases (HATs), histone deacetylases (HDACs), histone methyltransferases (HMTs) and histone demethylases (HDMs), as well as DNA methylation, caused by DNA methyltransferases (DNMT) (81). Histone acetylation is one of the key histone modifications, in which the expression of specific genes of the innate immune cells are regulated via histone acetylation, leading to a more open chromatin structure following stimulation, marked by H3K4m1 and H3K27ac signatures (82). This facilitates the binding of transcription factors and RNA polymerase to promotors of genes and initiates their expression (e.g., increased histone acetylation in pro-inflammatory genes induces the inflammatory molecules that are involved in controlling an infection) (82, 83). Histone methylation is another epigenetic modulation involved in trained immunity. Certain histone methylations might either activate or repress the expression of genes perturbed during trained immunity in innate immune cells. For example, increased trimethylation of histone H3 at lysine 4 (H3K4me3) gene associated with trained immunity, contributes to a stronger proinflammatory immune response against Mtb (84). The changes in histone modifications associated with trained immunity generate epigenetic memory that persists over time and allows the innate immune cells to recall previous encounters related to specific pathogens or stimuli. Epigenetic memory enables the cells to respond rapidly by promoting the expression of crucial genes that are necessary for an effective immune response (82, 85). The reprogramming of innate immune cells by various stimulants such as BCG and β-glucan via regulating epigenetic/histone modifications is initiated by downstream host signaling upon engagement of cellular receptors with PAMPs and DAMPs (18, 86). Recently, cytokines and chemokines including IL-1β, GM-CSF and M-CSF have emerged as inducers of epigenetic remodeling and are implicated in the training of innate immune cells (28, 87, 88). The mechanism of trained immunity activation includes immunometabolic reprogramming of the innate immune cells, which is also associated with changes in epigenetic regulation (18, 86). Some of the histone modifications associated with trained immunity are discussed below:

A). Histone acetylation catalyzed by HATs, leads to relaxation of chromatin structure, allowing for increased accessibility of transcription factors to gene promoters. Enhanced histone acetylation at the promoters of cytokine and chemokine genes, such as TNF-α, IL-6, and CXCL8, facilitates their transcription. Increased acetylation of histone H3 and H4 at specific gene loci promotes the assembly of transcriptional activators and coactivators, such as NF-κB and AP-1, leading to robust and sustained expression of inflammatory mediators (6–10, 18).

B). Histone methylation can occur on lysine (Lys) and arginine (Arg) residues, leading to either activation (e.g., H3K4me3) or repression (e.g., H3K9me3, H3K27me3) of gene expression depending on the site and degree of methylation. Increased trimethylation of histone H3 at lysine 4 (H3K4me3) is associated with transcriptional activation and is enriched at the promoters of actively transcribed genes, including pro-inflammatory cytokines and chemokines. Increased levels of H3K4me3 at specific gene loci, such as those encoding TNF-α, IL-6, and CXCL8, promote their transcriptional activation and contribute to the heightened inflammatory response in trained immune cells. Conversely, histone methylation at repressive marks, such as H3K9me3 and H3K27me3, is reduced at pro-inflammatory gene loci, allowing for their transcriptional activation and sustained expression.

C). DNA methylation involves the addition of a methyl group to cytosine residues within CpG dinucleotides, typically resulting in transcriptional repression when located in gene promoters. Trained immunity is associated with dynamic changes in DNA methylation patterns, including both hypomethylation and hypermethylation events. Hypomethylation of immune-related gene promoters, such as those encoding cytokines (e.g., TNF-α, IL-6) and pattern recognition receptors (e.g., TLRs), facilitates their transcriptional activation in trained immune cells. Conversely, hypermethylation of genes involved in negative regulators of inflammation, such as SOCS1 and SOCS3, may contribute to the sustained pro-inflammatory phenotype in trained cells.

These epigenetic modifications support a heightened state of readiness by promoting transcriptional activation of genes and priming innate immune cells to rapidly produce pro-inflammatory cytokines and chemokines, while dampening the expression of negative regulators, thereby enhancing immune responses upon re-stimulation and/or infection (6–10, 18).

#### 2.3.3 Aging and trained immunity regulation

Age-related changes in the epigenetic landscape, including changes in DNA methylation patterns, histone modifications, and chromatin structure. can affect the expression of genes involved in the induction and maintenance of trained immunity (89). Alterations in histone modifications and chromatin accessibility may impact the ability of immune cells to undergo epigenetic reprogramming in response to training stimuli, leading to differences in trained immunity outcomes between younger and older individuals (90). Similarly, aging influences the metabolic reprogramming of immune cells during trained immunity (89). For example, alterations in nutrient availability or mitochondrial dysfunction may impair the ability of immune cells to switch metabolic pathways and support enhanced responsiveness. Dysregulation of metabolic pathways, such as glycolysis, OXPHOS, and fatty acid metabolism, in aged immune cells can affect their energy production, biosynthetic capacity, and functional responses, potentially impacting trained immunity. Furthermore, the age-associated changes in epigenetic and metabolic regulation of immune cells can contribute to immune dysfunction, referred to as immunosenescence, characterized by reduced immune cell function, affecting the induction and maintenance of trained immunity and impaired responses to vaccination, and increased susceptibility to infections and inflammatory diseases (91). Thus, understanding the impact of age on epigenetic and metabolic pathways involved in trained immunity is essential for developing age-specific strategies to enhance immune responses in older individuals, potentially improving vaccine efficacy, host defense, and immune health in aging populations (91).

#### 2.3.4 Metabolic changes associated with trained monocytes and macrophages

**Figure 3:**
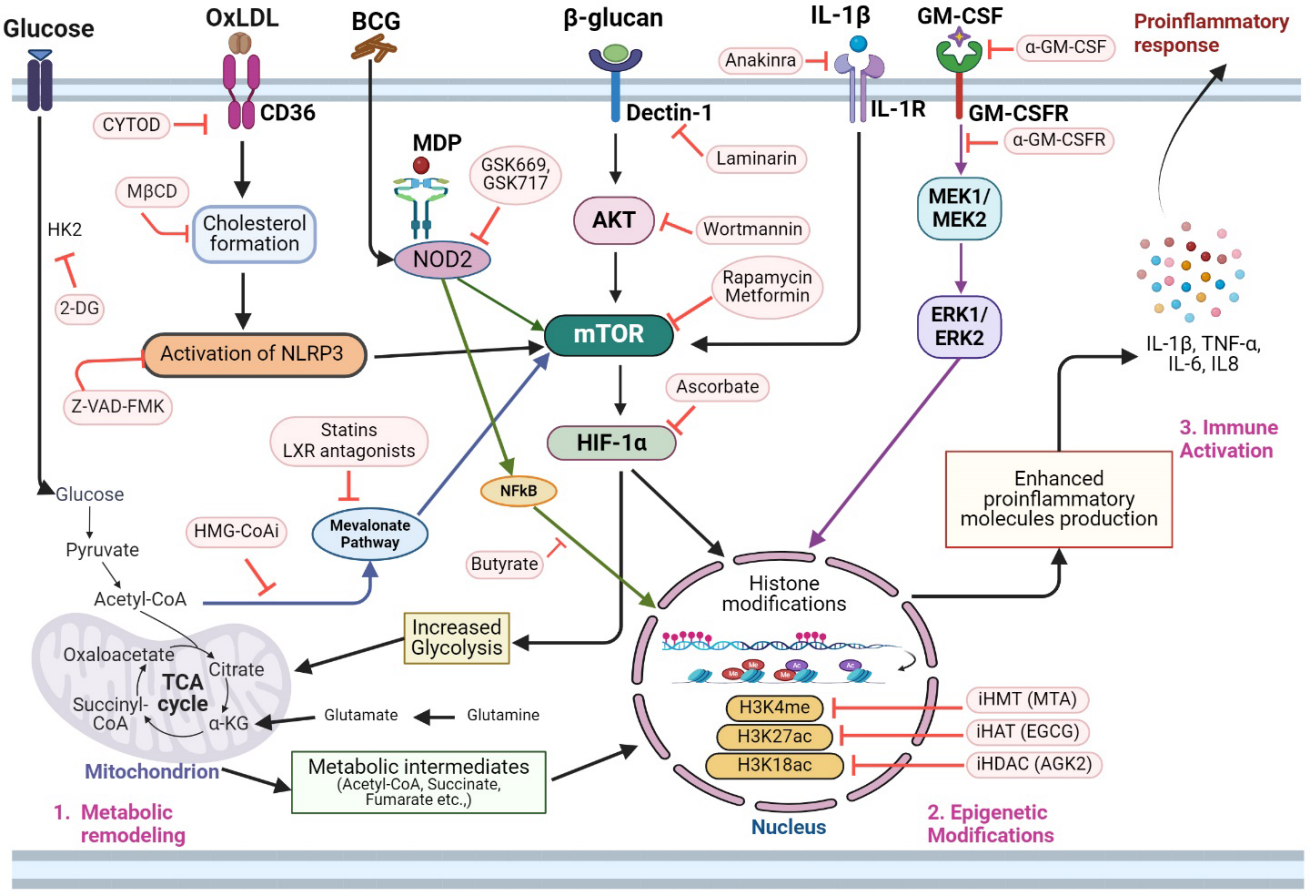
Integrated molecular metabolic and epigenetic network adaptations and potential interventional targets of trained immunity. Activation of trained immunity in innate immune cells involves significant changes in glycolysis, TCA cycle, fatty acid, and amino acid metabolism. 1. The metabolic remodeling in trained innate immune cells involves increased glycolysis and the production of several metabolic intermediates of glycolysis, which participate in the TCA cycle, and mevalonate pathway. Thus, inhibition of glycolysis pathway enzymes (e.g., HK2 inhibition by 2-DG) can dampen the overall metabolic and epigenetic modulations needed for immune activation of trained immunity. OxLDL is involved in cholesterol formation and subsequently in the activation of the NLRP3 inflammasome pathway, thus, activating trained immunity. The latter pathway also impacts mTOR signaling. Therefore, inhibition of the oxLDL-CD36 pathway using CYTOD or inhibition of cholesterol synthesis by MβCD2 can impair NLRP3 activation. Additionally, Z-VAD-FMK can directly inhibit NLRP3 and mTOR activation pathways and reduce trained immunity. Metabolites of the integrated carbohydrate (e.g, glycolysis) and amino acid (glutamine-glutamate) network also play crucial roles in regulating epigenetic modifications such as histone acetylation (acetyl-CoA), histone demethylation (αKG) and H3K4Me3/H3K27Ac expression (fumarate). Inhibition of HMG-CoA (HMG-CoAi) blocks the channeling of acetyl-CoA into the mevalonate pathway, which can also be blocked by statins and LXR antagonists. Since the mevalonate pathway upregulates trained immunity through epigenetic remodeling, treatment with HMG-CoAi and statins can reduce trained immunity activation. Dectin-1 and NOD2 are key receptors that activate trained immunity upon binding with β-glucan and MDP, respectively. Since activation of these pathways leads to both metabolic and epigenetic reprogramming, inhibition of the Dectin-1 pathway with laminarin or wortmannin, as well as inhibition of the NOD2 signaling with GSK669 or GSK719 or butyrate downregulates trained immunity activation. Similarly, the blockade of mTOR and HIF1α with rapamycin, metformin, or ascorbate, respectively, negatively affects trained immunity activation. In addition to PAMPs, host-derived molecules such as IL-1β and GM-CSF can induce metabolic rewiring and histone modifications through IL-1R or MEK/ERK-mediated signaling, respectively, during trained immunity. Therefore, antibodies that target these cytokines/chemokines can dysregulate immune activation through interference with metabolic, epigenetic, and immune activation pathways in trained cells. Similarly, blocking of specific molecules involved in histone acetylation (e.g., H3K18ac and H3K27) and histone methylation (H3K4me) pathways can alleviate the epigenetic changes needed to activate proinflammatory gene expression in trained cells. 3. Activation of integrated metabolic, epigenetic, and immune function pathways in trained cells ensures both the energy and chromatin accessibility that is needed for an elevated proinflammatory and antimicrobial molecule production by the innate immune cells. 2DG, 2-deoxy-d-glucose; HK2, hexose kinase-2; oxLDL, oxidized low-density lipoprotein, CYTOD, cytochalasin D; MβCD, methyl-β-cyclodextrin; HMG-CoAi, hydroxy-methyl-glutaryl-coenzyme A reductase; Dectin-1, C-type lectin receptor dectin-1; NOD2, nucleotide-binding oligomerization domain-containing protein 2; MDP, muramyl dipeptide; mTOR, mechanistic target of rapamycin; HIF1α, hypoxia-inducible factor 1α; AKT, Ak strain transforming; MEK, mitogen activated protein kinase kinase; ERK, extracellular signal regulated kinase; NFκB, nuclear factor kappa-B; GM-CSF, granulocyte–macrophage colony-stimulating factor; H3K18ac, H3K18 acetylation; H3K27ac, H3K27 acetylation; H3K4me3, H3K4 trimethylation; HATi, histone acetyl transferase inhibitor; EGCG, epigallocatechin-3-gallate; HDACi, histone deacetylase inhibitor; AGK2, a sirtuin-2 inhibitor; HMTi, histone methyltransferase inhibitor; MTA, methylthioadenosine; IL1β, interleukin 1 beta; TCA cycle, tricarboxylic acid cycle; αKG, alpha-ketoglutarate. The image was created in BioRender.

Trained immunity causes not only epigenetic reprogramming but also rewires metabolic pathways such as glycolysis, tricarboxylic acid (TCA) cycle, and lipid and amino acid metabolism of the trained innate immune cells (**Figure 3**). The combined effect of epigenetic and transcriptional modulations of genes in trained cells also underpins the altered metabolic state, such as activation of glycolysis shift and an elevated release of pyruvate, which is the end product of glycolysis (92). As per the energy requirement of the trained macrophages, the pyruvate might enter either into OXPHOS or the TCA cycle, although the former pathway relies on the latter.

##### 2.3.4.1 Glycolysis, OXPHOS and TCA cycle

Since activated host cells consume more glucose than resting cells, glycolysis is activated during training/stimulation of the innate immune cells to meet the energy demand to serve a proinflammatory role (93). Although glycolysis is not an efficient way for the cell to generate ATP, this process can rapidly be induced upon stimulation of the innate immune cells (94). The primary function of the glycolysis cycle is to break glucose molecules to produce ATP and release pyruvate, which is further transformed into acetyl CoA and participates either in the TCA or fatty acid oxidation (FAO) cycle. Importantly, glycolysis is activated in trained innate immune cells independent of the stimulus; thus, BCG, β-glucan, and lipoproteins can induce the glycolytic pathway (93, 95). Studies report that stimulants like β-glucan, BCG, and lipoproteins can induce glycolysis in innate immune cells (96). Two independent studies on stimulation of mice with β-glucan reported the induction of immune mediators such as IL-1β and GM-CSF with increased glycolysis, and mainly in trained monocytes it is reported that pyruvate is converted to lactate (97). The persistent activation of glycolysis is regulated by a key transcriptional regulator, namely the hypoxia-inducible factor-1 alpha (HIF1α), which is stabilized by succinate, an intermediate metabolite of glycolysis. Furthermore, succinate and fumarate can act as epigenetic modulators for antagonizing histone or DNA methylation and facilitating long-lasting expression of genes involved in the glycolysis pathway (94, 95). The increased glycolysis impacts the mammalian target of rapamycin (mTOR) and HIF-1α pathway representing the metabolic basis of trained immunity (96). Recent studies on HIF-1α knockout mice reported that the absence of these specific pathways, impacted the trained immunity at the epigenetic level and abrogated proinflammatory cytokine production (96).

OXPHOS is a more efficient but slow process to produce cellular energy, which involves the mitochondrial electron transport chain complexes that convert succinate or fumarate to release ATP. In general, glycolysis and OXPHOS operate in opposite directions, such that activation of the former dampens the latter and vice versa (98–100). It has been reported that β-glucan can induce OXPHOS shift, resulting in a higher intracellular ratio of NAD^+^ to its reduced form NADH, thus producing more ATP (97, 101, 102). However, recent findings suggest that trained innate immune cells can activate both glycolysis and OXPHOS pathways at the same time (94) to serve as cellular energy sources to meet the demands of activated/trained cells (103). In a study by Arts et al., the stimulation of macrophages with β-glucan and BCG upregulated the expression of glycolytic enzymes with an increase in NAD^+^ and NADH ratio (104).

TCA is a crucial metabolic cycle of the cell as it oxidizes the glycolysis substrates. Trained macrophages exhibit a higher level of oxygen consumption with a decrease in the use of OXPHOS (104). Although OXPHOS is decreased, the TCA cycle is not completely inactive; rather, the metabolites of the TCA cycle such as succinate, fumarate, and citrate exist at a higher level in trained cells compared to non-trained immune cells (104). Additionally, increased/accumulated TCA metabolites can serve as a key factor for fatty acid (FA) synthesis. Citrate induces gluconeogenesis and lipid metabolism pathways by inhibiting the glycolysis and TCA cycle (105). Stimulation with β-glucan increased the levels of TCA cycle metabolic intermediates, succinate, and fumarate, in trained monocytes and macrophages, with increased glycolysis and IL-1β production through HIF-1α pathway (106). Furthermore, fumarate accumulation blocked the function of HDMs and induced epigenetic modifications in βglucan-trained monocytes, which activated the proinflammatory response of these cells (107). Together, these studies demonstrate that carbohydrate metabolism, including glycolysis, OXPHOS, and TCA cycle are critical primary metabolic components of trained immunity, which is linked with the immune activation of innate immune cells upon stimulation with PAMPs and DAMPs, through alterations in epigenetic reprogramming.

##### 2.3.4.2 Lipid metabolism

Lipid metabolism is interconnected with carbohydrate and amino acid metabolism. For example, citrate, which is a common metabolite produced from the citric acid cycle, is converted into acetyl CoA, which enters lipid metabolism or cholesterol synthesis pathway and accumulates as stored fatty acids (108). However, the organelles where the fatty acids are stored generate proinflammatory responses related to trained immunity by degrading the stored lipids to restore acetyl CoA through β-oxidation. In trained innate immune cells, the homeostasis of cholesterol and FA metabolism is mediated by the liver X receptor (LXR) (109). *In vitro* studies using human monocytes trained with BCG or Tri-palmitoyl-S-glyceryl-cysteine (Pam3cys), a TLR-2/6 pathway agonist, and LXR agonist were reported to induce intracellular acetyl-Coa levels, accompanied by histone modification and increased pro-inflammatory responses through activation of IL-1β signaling (109). In another study, mevalonate, a metabolite of the cholesterol pathway, was shown to induce trained immunity by activating mTOR and IGF1-R pathways and related histone modification involved in inflammation; inhibition of mevalonate pathway using statins prevented trained immunity in these myeloid cells (110). Importantly, the trained immunity phenotype is constitutively activated in patients with hyperimmunoglobulin D syndrome, who accumulate mevalonate due to a defective mevalonate degradation pathway (110). In mice treated with β-glucan, induction of cholesterol and lipid metabolism was noted in myeloid progenitor myeloid cells (96, 111). Furthermore, modulation of lipid metabolism in the myeloid progenitor cells during trained immunity was associated with the expansion of these cells and change in their phenotype skewed towards proinflammatory responses (96, 111). Training of mice HSC with LPS was shown to upregulate the expression of genes involved in FAO and OXPHOS (31, 61, 86, 112). Furthermore, cholesterol metabolism was reported to be indispensable for trained immunity activation in monocytes upon β-glucan induction and inhibition of cholesterol metabolism reduced the β-glucan-induced trained immunity in mice (107, 113). Additionally, inhibition of hydroxy-3-methylglutaryl CoA reductase, a key enzyme in the cholesterol synthesis pathway, negatively impacted the trained immunity established with β-glucan stimulation *in vitro* (113). Some of the lipid metabolism signaling pathways that impact trained immunity are as follows:

***A).*** *Peroxisome proliferator-activated receptors (PPARs).* PPARs are nuclear receptors that regulate lipid metabolism and inflammation (114). Activation of PPARγ promotes lipid uptake, storage, and adipogenesis, which can modulate the metabolic state of trained cells and impact their inflammatory responses. Pharmacological activation of PPARγ has been shown to enhance trained immunity, leading to increased cytokine production and antimicrobial activity in macrophages (115).
*B). Sterol regulatory element-binding proteins (SREBPs).* The SREBPs are transcription factors that regulate lipid metabolism and cholesterol biosynthesis signaling pathways, which are essential for membrane integrity and cellular functions (116). SREBPs are activated by TLR4-mediated innate signaling, which upregulates caspase-1-mediated IL-1b production by macrophages (117). Since SREBP signaling integrates hypoxia, autophagy, phagocytosis and antimicrobial response in innate immune cells, dysregulation of this pathway can alter lipid metabolism and inflammatory responses in trained cells (116, 117).
*C). The mechanistic target of rapamycin (mTOR)* is a central regulator of cellular metabolism, growth, and immune responses. mTOR signaling integrates signals from nutrient availability, energy status, and growth factors to modulate cellular responses (118). During training, activation of mTOR signaling promotes glycolysis, lipid biosynthesis, and protein synthesis, supporting the metabolic demands of activated immune cells. Thus, modulation of mTOR activity/signaling can influence trained immune cell metabolism and functions (119).
*D). Signaling pathways activated by fatty acids*, such as TLR signaling and inflammasome activation, can influence immune responses and trained immunity (120). Lipid mediators derived from fatty acids, such as prostaglandins, leukotrienes, and resolvins, regulate inflammation and immune cell activation, potentially modulating trained immunity outcomes (91, 120). Similarly, cholesterol metabolism influences both NLRP3-dependent and independent inflammasome activation and proinflammatory cytokine (e.g., IL-1β) production in innate immune cells (121).

##### 2.3.4.3 Amino acid metabolism

Amino acids, such as methionine, glutamine (Gln), proline (Pro), and aspartate (Asp) play a key role in stimulating and activating immune cells. Upon stimulation with agents such as BCG or β-glucan, innate immune cells undergo metabolic reprogramming to meet the increased energy demands associated with enhanced effector functions. Acquisition of amino acids, driven by specific cellular transporters, is crucial for cell function; for example, transportation of methionine stimulates T-cell activation (122). Similarly, Gln, which is present in the mitochondrial outer matrix serves as a source of succinate, fumarate, and citrate involved in the TCA cycle, and serves as a vital energy source for immune cells (123). In trained immune cells, Gln metabolism influences the activation of immune cells, while activation of HIF-1α by various stimulants, including BCG and β-glucan, increases Gln metabolism, leading to an elevated α-ketoglutarate (α-KG) level, which is channeled into the TCA cycle for energy production (113). Consistently, pharmacological inhibition of Gln metabolism attenuated the trained immunity phenotype in β-glucan-stimulated macrophages (113). Furthermore, H3K4me3, a chromosome modification that impacts gene transcription has been reported to be reduced when Gln uptake was blocked in breast cancer cells (124).

Neonatal mice infected with Pneumonia virus of mice (PVM, which is pathogenically similar to the respiratory syncytial virus [RSV] that affects humans) and stimulated with ovalbumin, have established trained immunity with increased Pro biosynthesis in alveolar macrophages (125). In addition, Asp was shown to be highly induced in trained macrophages compared to non-trained cells. Asp metabolism is also crucial for other metabolic cycles such as the urea cycle, gluconeogenesis, and purine synthesis, all of which contribute to activated cellular processes during trained immunity (97, 126). In macrophages stimulated with LPS and IFNγ, the level of Asp metabolites, including asparagine was shown to be dampened (127). In these proinflammatory macrophages, Asp metabolism elevated the secretion of IL-1β through the activation of HIF-1α pathway. Furthermore, supplementation of Asp elevated the inflammatory response of macrophages in treated mice and piglets (127). The consumption of methionine, which is the precursor of S-adenosylmethionine (SAM) involved in histone methylation, is elevated in monocytes trained with β-glucan (113). Thus, methionine can impact the regulation of gene expression through SAM during the training of innate immune cells.

Some of the amino acids influence the trained immunity through multiple, integrated mechanisms (92, 128, 129). For example, Gln and Arg serve as a precursor for nucleotide synthesis, and polyamine synthesis, respectively, supporting rapid DNA replication and cell proliferation of trained cells. Gln also fuels the TCA cycle and OXPHOS, providing energy for activated trained cells. Finally, Gln and cysteine (Cys) are, respectively, a substrate and precursor for glutathione synthesis, which contributes to the antioxidant defenses, redox balance, and protection of trained cells from stress, while Arg is a precursor for nitric oxide (NO) production by inducible nitric oxide synthase (iNOS or NOS2) in innate immune cells; NO plays a crucial role in immune signaling and antimicrobial response of trained cells. Similarly, tryptophan (Trp) is the precursor to serotonin and kynurenine synthesis, which have immunomodulatory effects, including immune activation and immune tolerance. Finally, leucine (Leu) is an essential amino acid that activates the mTOR signaling pathway in trained cells, which promotes protein synthesis, cell growth, and proliferation, supporting cell activation and effector functions (92, 128, 129). Thus, altering the availability of specific amino acids through supplementation can have an immunomodulatory effect on trained cells, which impacts the inflammatory response and antimicrobial response of these cells (92, 128, 129).

#### 2.3.5 Interdependence of immunologic, epigenetic and metabolic mechanisms of trained immunity

The immunologic mechanism of the trained immunity involves the reprogramming of innate immune cells, marked by changes in gene expression and functional alterations in immune cells, leading to increased cytokine production, antigen presentation, and antimicrobial activity of trained cells to exhibit enhanced responsiveness to subsequent antigen/pathogen challenges (**Figure 3**). For example, data from *in vitro* and *in vivo* studies have shown that training of APCs by BCG, β-glucan and/or LPS leads to increased production of proinflammatory cytokines such as TNF-α, IL-1β and IL-6 (6–10, 18). Trained proinflammatory APCs also display enhanced aerobic glycolysis and OXPHOS to meet the increased energy needs (74–78). Importantly, stimuli that induce trained immunity also impact epigenetic reprogramming, which alters the expression of genes involved in pro-inflammatory and metabolic responses of innate immune cells. For example, trained immunity modulates specific histone modifications such as H3K4me and H3K27ac, which can open-up the chromatin and facilitate the induced expression of pro-inflammatory cytokines (TNF-α, IL-1β and IL-6). Similarly, changes in chromatin structure facilitate the expression of antimicrobial genes in trained immune cells (68–72). Similarly, metabolic reprogramming is closely intertwined with immunological and epigenetic changes during trained immunity. Activation of immune cells by trained immunity requires substantial energy and metabolic resources, and different metabolic pathways, such as glycolysis, OXPHOS, and fatty acid metabolism, can impact immune cell function (6–10, 18). Various stimuli that induce trained immunity can promote metabolic shifts, such as increased glycolysis, glutaminolysis, cholesterol and fatty acid metabolism in innate immune cells. These metabolic pathways generate intermediary molecules, which regulate epigenetic remodeling and proinflammatory cytokine production in trained cells (6–10, 18). For instance, metabolites such as acetyl-CoA, α-KG, and S-adenosylmethionine (SAM) act as cofactors for histone acetylation, histone methylation, and DNA methylation, respectively (6–10, 18). On the other hand, epigenetic modifications can influence metabolic pathways by regulating the expression of metabolic enzymes and transporters. For example, histone acetylation can activate the mTOR-HIF-1α-pathway-mediated upregulation of aerobic glycolysis, OXPHOS, and fatty acid metabolism in trained cells (6–10, 18). Thus, the pathways of proinflammatory response, and epigenetic modifications, including changes in DNA methylation, histone modifications, and chromatin accessibility, as well as metabolic rewiring, are intertwined and play a central role in the regulation of trained immunity (6–10, 18). This cross-talk between metabolic shifts and epigenetic changes ensures a coordinated and integrated immune response in trained cells. While metabolic reprogramming provides the energy and substrates necessary for immune cell activation, epigenetic modifications fine-tune gene expression to enhance cytokine production, antigen presentation, and antimicrobial activity (6–10, 18). This synergy between immune response, metabolism, and epigenetics allows trained cells to mount a more robust and sustained immunological response upon re-exposure to pathogens, contributing to the enhanced protective effects of trained immunity (6–10, 18).

#### 2.3.6 Durability of trained immunity

The durability of the immune response in trained cells is regulated at the level of hematopoietic progenitor stem cells (HSPCs) in the bone marrow (18). For example, exposure of BCG to the bone marrow resulted in epigenetic and transcriptional changes of HSPCs, leading to elevated myelopoiesis and conferring better protection of mice against TB (130). Trained immunity involves the establishment of positive feedback loops that sustain the enhanced expression of proinflammatory cytokines and chemokines over time. Epigenetic modifications, such as H3K4me3 and H3K27ac marks, at enhancer regions of cytokine genes, facilitate the binding of transcription factors and RNA polymerase II, ensuring persistent transcriptional activation (6–10, 18). Similarly, metabolic memory, characterized by the accumulation of metabolic intermediates of glycolysis and OXPHOS and mitochondrial ROS, maintains the trained immunity phenotype and facilitates rapid recall responses of innate immune cells upon re-stimulation and/or microbial infection (6–10, 18). Some of the mechanisms underlying the dose-dependent response of trained immunity include the following:

*A). Intensity of stimulation*, such as the concentration of microbial components or cytokines, determines the extent of immune cell activation and subsequent epigenetic and metabolic reprogramming (6–10, 18). Low to moderate doses of the stimulus typically induce trained immunity, characterized by enhanced responsiveness and prolonged memory-like effects in innate immune cells. High doses of the stimulus, on the other hand, may lead to immune tolerance, where immune cells become desensitized and exhibit reduced responsiveness to subsequent challenges.
*B)*. *Epigenetic modifications*, such as histone acetylation, methylation, and DNA methylation are influenced by the intensity of the stimulus in innate immune cells. Moderate stimulation induces specific epigenetic changes that promote the expression of pro-inflammatory genes and enhance immune responses, leading to trained immunity. High-intensity stimulation may result in global changes in chromatin structure or DNA methylation patterns that suppress immune gene expression and induce immune tolerance (6–10, 18).
*C). Metabolic Reprogramming* is also impacted by the intensity of the stimulus, with moderate stimulation promoting metabolic pathways that support immune cell activation and effector functions. Low to moderate doses of the stimulus typically induce metabolic shifts towards glycolysis, pentose phosphate pathway, and glutaminolysis, providing energy and substrates for cytokine production and antimicrobial activity (6–10, 18). High doses of the stimulus may overwhelm metabolic capacity or lead to metabolic exhaustion, impairing immune cell function and promoting immune tolerance.

While specific thresholds for stimulation that distinguish between training and tolerance may vary depending on the context and experimental model, there are general trends observed. Moderate doses of the stimulus, falling within an optimal range, typically induce trained immunity, whereas low doses may not provide sufficient activation, and high doses may induce tolerance (6–10, 18). The threshold for stimulation may also depend on the sensitivity and responsiveness of innate immune cells, as well as the presence of regulatory mechanisms that modulate immune responses.

### 2.4 Trained immunity in neutrophils

Neutrophils (polymorphonuclear cells) are produced in the bone marrow and are the first responders to any injury or infection of the host. Although neutrophils have a shorter lifetime, they are the most abundant innate immune cells that provide significant and non-specific broad protection against related or unrelated pathogens (20, 131, 132). Although neutrophils are traditionally thought to lack long-term memory, recent studies suggest that they can retain epigenetic memory. Neutrophils are among the key effector cells of trained immunity with antigen-presenting functions, secreting cytokines that induce inflammatory factors, degranulate, and eliminate microbes through phagocytosis (133). A study reported that BCG vaccination in healthy individuals reprogrammed the neutrophils with increased expression of surface markers CD11b and CD66b, with concomitant dampening of CD62L and PDL1 expression, which persisted for at least 3 months, through epigenetic modifications (76). In this study, *in vitro* stimulation of blood-derived neutrophils, obtained after 3 months of BCG vaccination, with LPS or *Staphylococcus aureus* was shown to induce the expression of neutrophil activation markers CD11b and IL-8 (76). These trained neutrophils also showed increased degranulation and phagocytosis *in vitro* when stimulated with *Candida albicans*, Mtb, or LPS (76). Intranasal BCG vaccination of mice showed increased accumulation of neutrophils in the lungs, which helped to control subsequent Mtb infection by promoting antimicrobial responses (134).

In a zebrafish larvae model of *Shigella* infection, stimulation with BCG or β-glucan before infection elicited trained immunity in neutrophils, which showed epigenetic alterations, elevated ROS production, and antimicrobial responses (135). Importantly, induction of trained immunity in neutrophils has been shown to occur in a paracrine manner (136). In this study, treatment of neutrophils with soluble factors, secreted by mesenchymal stromal cells upon stimulation with CpG-ODN, a TLR-9 ligand, induced trained immunity marked by characteristic histone modifications and elevated granulopoiesis (136). Similarly, neutrophils isolated from mice treated with zymosan, which contains β-glucan, had elevated IL-6 levels upon ex vivo re-stimulation with LPS (35, 137). These trained neutrophils also showed elevated myeloperoxidase levels and improved killing of intracellular *L. monocytogenes* (35). Thus, these studies suggest that induction of trained immunity in neutrophils by various stimuli can promote neutrophil activation, which is useful for effective bacterial clearance (137). In contrast to these findings, subcutaneous vaccination of C57BL/6 mice with BCG was reported to control Mtb at 7 days post-infection through a non-trained immunity mechanism, involving neutrophils and macrophages (138).

Neutrophils, despite their short lifespan, inherit epigenetic modifications from the myeloid progenitor cells during hematopoiesis (101, 139). Neutrophils can undergo activation-induced epigenetic changes in response to antigenic stimulation or microbial challenge. These activation-induced epigenetic modifications can persist even after the stimulus is removed, allowing neutrophils to retain a primed/activated state and mount rapid and robust response upon re-stimulation (101, 139). For example, elevated H3K4me3 at the promoters of JAK/STAT signaling (e.g., STAT4), PI3K/AKT pathway as well as proinflammatory (IL-8, IL-1B) and metabolic (mTOR, HK1 and PFKB) network genes noted in the peripheral neutrophils of BCG-vaccinated individuals, compared to non-vaccinated controls (101). Induction of these pathways is the hallmark response of trained immunity (i.e., proinflammatory and antimicrobial responses), suggesting that histone methylation underpins the long-term trained response of neutrophils (101). Other epigenetic reprogramming markers, such as DNA methylation and histone modifications can be established during neutrophil development (granulopoiesis) and maintained throughout their lifespan in both circulation and at specific tissues (101, 139). Emerging evidence suggests that epigenetic modifications acquired by neutrophils in response to environmental cues or inflammatory signals may be transmitted to their progeny (139). For example, neutrophils can release extracellular vesicles containing epigenetic regulators such as microRNA and histones which may influence the landscape of neighboring cells or circulating progenitors (139). A recent study revealed an association between the epigenetic reprogramming of granulopoiesis as well as neutrophils and trained immunity induced by β-glucan in a murine model of cancer (140). In this study, mice treated with β-glucan displayed an anti-tumor phenotype mediated by trained neutrophils through a type I IFN signaling, and independent of the host adaptive immune response. Importantly, the trained immunity-mediated anti-tumor effect can be established in the naïve mouse upon adoptive transfer of neutrophils or transplantation of bone marrow from the β-glucan treated mice (140). These transgenerational epigenetic inheritance mechanisms could contribute to the persistence of epigenetic changes in neutrophils and their progeny, potentially contributing to immune memory across generations of immune cells.

### 2.5 Applications of trained immunity

**Figure 4:**
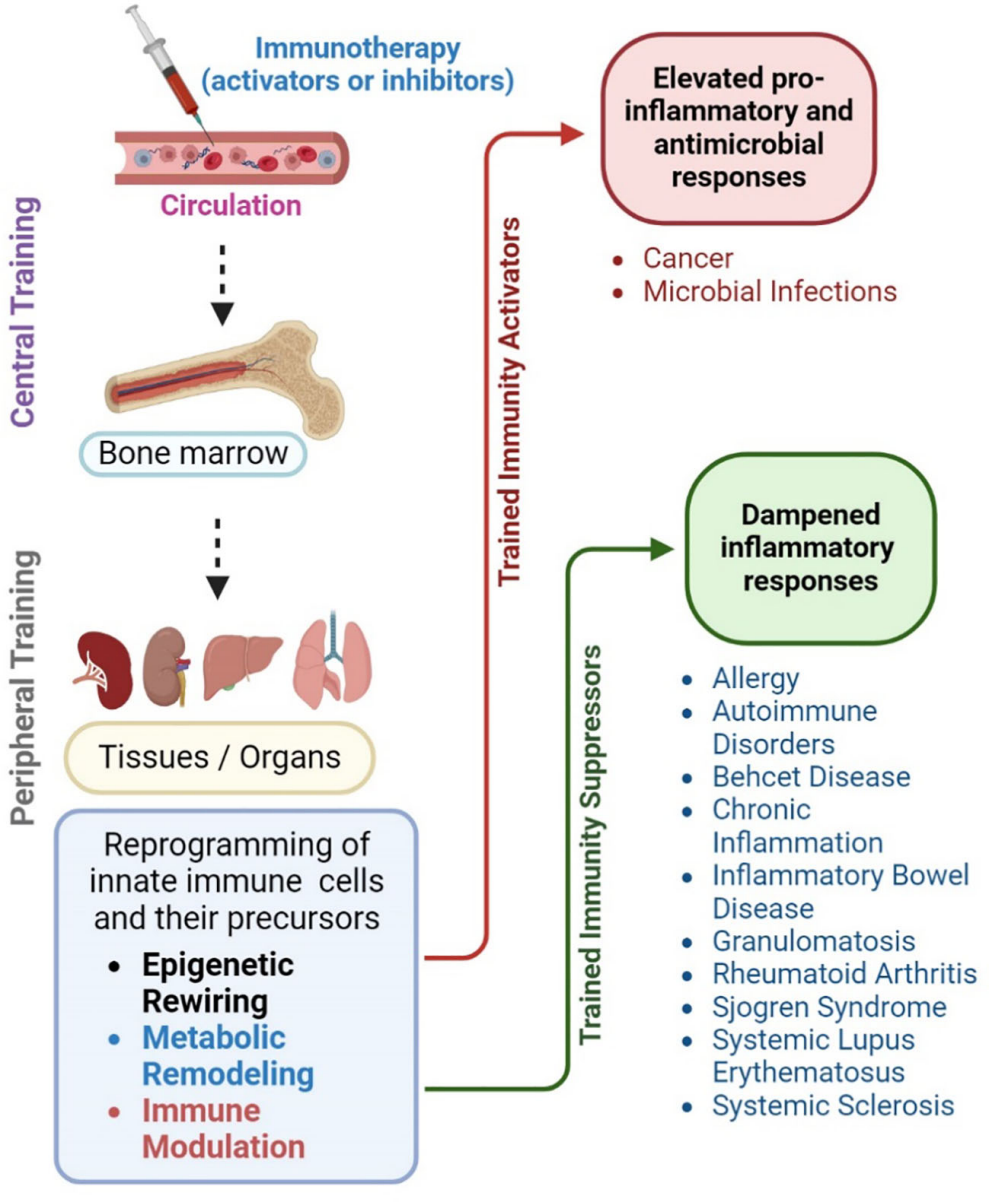
Potential of trained immunity for disease management. Trained immunity can be harnessed to devise novel intervention strategies to improve treatment for infectious and non-infectious diseases. For example, systemic administration of antigens (immunotherapy) that modulate trained immunity can mount a long-lasting central memory response among myeloid progenitor cells present in the bone marrow. These cells can reach various organs and tissues, where they can exert the features of trained immunity through epigenetic rewiring, metabolic remodeling, and immune activation upon encountering PAMPs or microbes. This peripheral training of innate immune cells results in increased proinflammatory and antimicrobial responses that can confer host protection in conditions such as cancer and infections, such as sepsis. Alternatively, the reprogramming of innate immune cells and their precursors can be suppressed or abolished by inhibiting specific pathways of epigenetic rewiring, metabolic remodeling, and immune activation. In this way, the exacerbated immune activation and inflammatory response due to trained immunity can be dampened. This approach is particularly useful to treat autoimmune disorders, allergies, systemic lupus erythematosus, systemic sclerosis, as well as chronic inflammatory diseases such as inflammatory bowel disease and rheumatoid arthritis. The image was created in BioRender.

Since trained immunity involves reprogramming of innate myeloid cells to induce robust and long-term efficient immune response upon pathogen encounter, several potential avenues of this pathway can be used as targets to develop intervention strategies (**Figure 4**). For example, trained immunity is regulated by epigenetic and metabolic changes in the innate immune cells relying on specific pathways and networks (18). Among the epigenetic modifications, histone methylation, acetylation, and DNA methylation are the key targets to tweak trained immunity. Drugs that target histone modification enzymes, such as HATs, HDACs, HMTs and HDMs can modulate the epigenetic landscape of trained cells and their effector functions (18). For example, HATs and H3K4Me3 activating drugs can promote histone acetylation and promoter access to enhance the production of proinflammatory (e.g., TNF-α, IL-6) and effector molecule (e.g., cathelicidins) production and the response of trained cells. Similarly, HDAC, HMT and DNMT inhibitors can be used, respectively, to influence histone methylation dynamics and enhance the stability and maintenance of DNA methylation marks to facilitate long-term memory-like responses associated with trained immunity. Metabolic pathways such as glucose metabolism and lipid metabolism, the shift in pentose phosphate pathway, aerobic glycolysis, and alterations in FAO might also be ideal targets to activate trained immunity (18). Increased glycolytic flux, fueled by glucose uptake and utilization, provides energy and biosynthetic precursors for enhanced effort functions and cytokine/chemokine production in trained cells. Furthermore, metabolic intermediates, such as α-KG, succinate and acetyl-CoA derived from metabolic pathways serve as substrates for epigenetic modifications and regulate the gene expression profile of trained cells. Thus, inhibitors and modulators of key enzymes of glycolysis or OXPHOS, as well as amino acid and fatty acid metabolism can be used to elevate the energy metabolism of trained cells for rapid and robust effector functions against pathogenic challenge. Furthermore, PRRs such as TLRs and nucleotide binding receptors are critical in recognizing the antigens and pathogens and interacting with trained immune cells. Transcription factors such as signal transducer and activator proteins (STAT) influence immune cell functions and proinflammatory signaling pathways such as IL-1β and IFN also might act as potential targets to enhance trained immunity.

Similarly, since trained immunity induces a broader but effective immune response, particularly against a range of pathogenic microbes, understanding the pathways and molecules involved in the activation of trained immunity can be useful in developing potential vaccine candidates against infectious diseases. Targeting pathways of trained immunity, such as histone modifications, metabolic reprogramming, and innate immune signaling, with specific agonists or antagonists cold enhance innate effector responses. This can be achieved by incorporating molecules, such as epigenetic or metabolic modulators that can induce trained immunity, into vaccine formulation. For example, incorporating small molecule agonists or adjuvants that promote trained immunity, such as β-glucan, BCG, or specific cytokines (e.g. IL-1β, IL-6) could be used to boost immune memory and improve vaccine efficacy. Similarly, adjuvants and live attenuated vaccines that can interact with PPRs, such as TLRs, NLRs and CLRs can augment vaccine-induced lasting immunity. These molecules act as strong adjuvants or immune stimulants during vaccine formulation and help in priming the immune system at a stronger level to encounter primary or secondary infection by divergent pathogens or in combating different types of cancer.

Since chemotherapy for cancer treatment is often highly cytotoxic, alternates such as immunotherapy have been sought as better treatment options. Importantly, the efficacy of immunotherapy can be improved by combining with agonists such as β-glucan, which can train and activate myeloid cells for better anti-tumor effector functions. This concept has recently gained attention in the treatment of neuroblastoma (NB) and metastatic pancreatic ductal adenocarcinoma (PDAC) treatment. For example, in a recent Phase II randomized clinical trial of patients with NB, adjunctive oral administration of β-glucan during bivalent, GD2 lactone/GD3 lactone-keyhole limpet hemocyanin conjugate vaccination was shown to increase the anti-GD2 IgG1 antibody titer without elevating toxicity, which was associated with better survival of vaccinated patients (141, 142). Moreover, an ongoing phase II clinical trial (Clinical trial NCT04936529) is evaluating the protective efficacy of this bivalent vaccine (OBT-821) combined with β-glucan as a dietary supplement and granulocyte-macrophage colony-stimulating factor (GM-CSF), against NB. The idea of including GM-CSF along with β-glucan is to increase the number of granulocytes, such as neutrophils by GM-CSF, while empowering those cells through β-glucan-mediated trained immunity to effectively control NB cells. Similarly, in a Phase II study (Clinical trial NCT00874848), BTH1677, a β-glucan immune modulator was shown to improve the efficacy of cetuximab, carboplatin, and paclitaxel as first-line treatment for non-small cell lung cancer (143). In addition, the tolerability and efficacy of β-glucan combined with a CD40 agonistic monoclonal antibody (CDX-1140) is being tested in a Phase 1b study on patients with PDAC (Clinical trial NCT04834778). The logic of this approach is that both CDX-1140 and β-glucan can promote the activation and maturation of APCs through non-redundant myeloid signaling pathways that shift the immune milieu of the tumor microenvironment (TME) and facilitate better clearance and control of cancer cells. Although the results of the PDAC trial are pending, these clinical studies indicate the potential application of trained immunity-based concepts to devise novel and improved treatment modalities for various diseases. It is worth noting that the cytokine/chemokine-induced trained immunity (e.g., IL-1b, GM-CSF, M-CSF) can potentially be useful as combination therapy in preventing/alleviating treatment-associated (e.g., chemo-/radio-/immune-therapy) or disease-associated complications. In a clinical study, treatment of GM-CSF in combination with rituximab and cyclophosphamide/doxorubicin/prednisone/vincristine improved the survival of patients with *de novo* diffuse large B-cell lymphoma (144). Similarly, G-CSF (e.g., pegfilgrastim) is already in clinical use for treating neutropenia occurring during the myelosuppressive chemotherapeutic regimen for cancer treatment (145). Thus, there is a higher potential for chemokines such as M-CSF to be used as an immune trainer (e.g. post-chemotherapy or after stem cell transplantation), which can induce epigenetic rewiring in HSCs *in vivo* (28), and thus may be helpful for disease management.

Trained immunity might be useful in designing vaccines against pathogens that can mutate or develop resistance over time; an activated innate memory response can effectively recognize and respond to the evolving strains. In addition, stimulators of trained immunity can be combined with traditional vaccines or immunotherapies to enhance persistent, longer host-protective immune responses. For example, epigenetic modulators, such as HDAC or HMT inhibitors, as well as metabolic modulators targeting glycolysis, OXPHOS can be combined with vaccines to improve the durability of trained immune response. Nutritional interventions, including dietary supplements such as vitamins, amino acids, or fatty acids, as well as mitochondrial-targeting antioxidants, can enhance the effectiveness of vaccines and boost immune memory. Thus, understanding individual variations in trained immunity responses, influenced by genetics, age, and environmental factors could be useful for developing personalized vaccines tailored to elicit a specific immune response. Moreover, biomarkers of trained immunity, such as epigenetic and metabolic signatures, and cytokine profiles, can be used to assess vaccine responsiveness and guide to improvise personalized vaccination strategies, particularly in vulnerable populations. Large-scale clinical studies are needed to identify genetic determinants, biomarkers, and other factors of variations in trained immunity. This approach would aid in developing personalized precision medicine based on trained immunity for the effective treatment of infectious and chronic diseases.

### 2.6 Limitations of trained immunity

Although the concept of trained immunity emerged recently, it has gained significant momentum in explaining the host response to microbial infections and chronic diseases, with the potential for clinical applications. However, trained immunity generates a non-specific, immune response towards unrelated pathogens, compared to the antigen-specific, targeted response of adaptive immunity. This lack of specificity might result in an overstimulated immune system causing unnecessary inflammation and tissue damage. Unlike adaptive immunity, trained cells do not discriminate between pathogen-specific antigens, this lack of antigen specificity could lead to non-specific or inappropriate immune responses. In addition, the duration and persistence of trained immunity are yet to be fully unraveled as it is unclear how long the enhanced protective immunity would last and whether it would have any long-term side effects. The non-specific nature of trained immune cells also raises the possible risk of the immune system targeting self-antigens, mistakenly leading to immunopathology, autoimmunity, or chronic inflammatory conditions. In this scenario, the identification of targetable negative regulators or checkpoints that modulate trained immunity pathways and maintain immune homeostasis would reduce the risk of autoimmune reactions. Furthermore, designing newer vaccines based on trained immunity might be challenging, due to non-specificity, as traditional vaccines are designed to induce antigen-specific adaptive immune responses, and attempting to replicate this specificity with a trained immunity concept might be complex and complicated. Therefore, understanding the interconnectedness of pathways/networks involved in the molecular and cellular processes of trained immunity, including epigenetic modifications, metabolic reprogramming, and immune response, is vital for identifying specific, context-dependent intervention targets. Moreover, the effector and regulatory functions of trained immune responses can vary significantly between individuals and populations based on factors such as age, sex, prior exposure to microbes, and genetic variations, which makes it difficult to predict and control the trained immune responses as it leads to hyperinflammation. Therefore, accounting for this heterogeneity and developing personalized approaches, tailored to individual immune profiles may be necessary for optimizing clinical outcomes. The duration and persistence of trained immune responses may vary, and the longevity of memory-like responses is currently not well understood. Therefore, studies on the molecular pathways and epigenetic mechanisms governing the durability of trained memory and enhancing memory persistence are urgently needed to devise improved interventions for long-lasting immune protection.

### 2.7 Ethical and regulatory considerations

Ethical considerations pose an additional challenge in implementing interventions based on trained immunity, as inducing a non-specific immune response, without understanding the long-term effects is unlikely to be accepted by the community. Due to the potential off-target effects of interventions targeting trained immunity, ensuring the safety of such interventions and minimizing adverse effects is critical for clinical translation, particularly in vulnerable populations. Thus, designing clinical trials to evaluate the safety and efficacy of interventions targeting trained immunity poses unique challenges. Identifying appropriate biomarkers of trained immunity, defining clinically relevant endpoints, and conducting long-term follow-up studies are essential for patient stratification, treatment selection, assessing therapeutic efficacy, and establishing clinical utility. In this regard, collaboration between basic scientists, clinicians, immunologists, pharmacologists, and other stakeholders is essential for advancing translational research efforts. Integrating expertise from this multidisciplinary group can accelerate the translational research to clinical applications. In addition, compliance with regulatory approval, including patient consent, privacy, data sharing, and protection of personal data, per ethical guidelines is essential for advancing clinical translation efforts. Patient-centered approaches that prioritize patient needs, preferences, and values through the engagement of patients, caregivers, and advocacy groups that balance scientific rigor and patient welfare, can enhance awareness and support for trained immunity-based therapies.

## 3 Summary and conclusion

This review underscores the transformative potential of trained immunity in immunology, paving the way for novel therapeutic strategies that leverage innate immune memory. In summary, trained immunity encompasses the immunologic determinants of both classical innate and adaptive responses, elicited in innate immune cells. Several clinical observations involving vaccine-induced immune-boosting of the host that conferred broad protection against a range of pathogens were attributed and/or explained, at least in part, by trained immunity. Recently, the molecular mechanistic aspects of trained immunity regulation upon stimulation with various homogeneous or heterogeneous stimulants have been actively investigated. Different stimuli trigger varied trained immune responses, influencing disease resistance and immune health. These stimuli, including microbial components and vaccines, induce distinct changes in innate immune cells, enhancing their responsiveness upon re-exposure. Thus, although trained immunity confers protection against infections, it may also contribute to chronic inflammatory conditions. Therefore, understanding and modulating trained immunity offer therapeutic potential for improving vaccine efficacy and treating diseases. However, individual variability necessitates personalized approaches to optimize immune responses and health outcomes. Understanding the mechanisms of epigenetic persistence in myeloid cells and their implications for immune memory could provide insight into novel strategies for enhancing host defense and immune responses in health and disease. More research is needed to explore mechanisms regulating trained immunity to prevent excessive or aberrant immune responses. This would help in devising approaches to selectively activate pathways or immune cell subsets of trained immunity, minimizing non-specific off-target effects. Future research on the understanding of these mechanisms would aid in devising strategies to prolong the host-protective immunity elicited by trained immunity. Further, specific components of the trained immunity mechanisms, such as epigenetic and metabolic checkpoints may be harnessed for developing targeted interventions for infectious and non-infectious diseases in the future.

## Author contributions

GB: Data curation, Formal analysis, Investigation, Methodology, Visualization, Writing – original draft, Writing – review & editing. SS: Conceptualization, Formal analysis, Funding acquisition, Investigation, Project administration, Supervision, Writing – review & editing.
